# Supplementary material for: Extracellular Vesicles as Mediators of Nickel-Induced Cancer Progression
Source: Int J Mol Sci. 2022 Dec 17;23(24):16111. doi: 10.3390/ijms232416111 (PMC9785150; doi:10.3390/ijms232416111)
Supplement: Supplementary file 1 [file ijms-23-16111-s001.zip › ijms-2051127-supplementary.pdf]

Supplementary figures

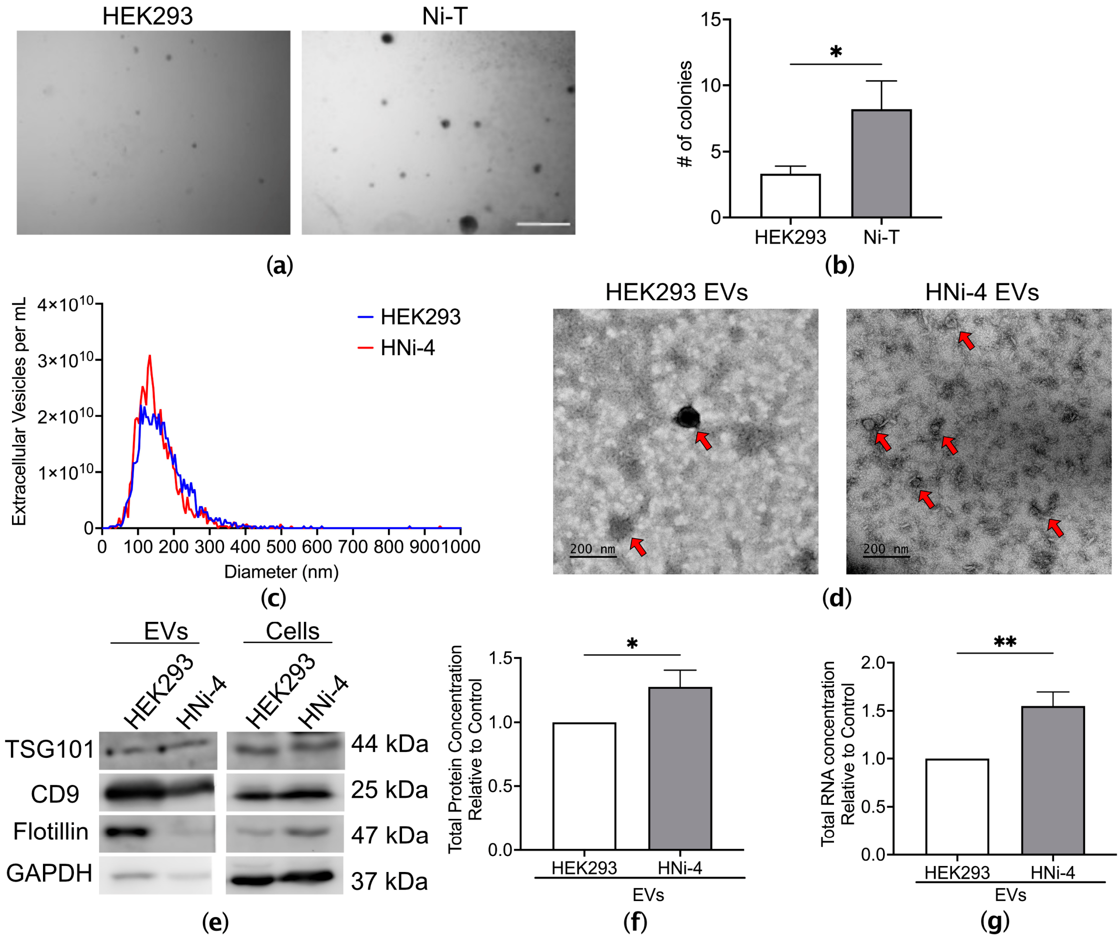

Figure S1. Characterization of EVs from Ni-transformed HEK293 cells. HEK293 cells were treated with 100 μM $NiCl_2$ for 4 weeks, and seeded in soft agar (**a-b**). EVs from HEK293 and #4 Ni-transformed HEK293 cells (HNi-4) by UC and were characterized by several methodologies including (**c**) nanoparticle tracking analysis (NTA). (**d**) transmission electronic microscopy (TEM) and (**e**) immunoblot of EV markers CD9, flotillin, and TSG 101.Red arrow: extracellular vesicles. Comparison of total protein (**f**) and total RNA (**g**) of EVs from HEK293 and HNi-4. Values are presented as mean ± SEM. $*p < 0.05$, $**p < 0.01$.

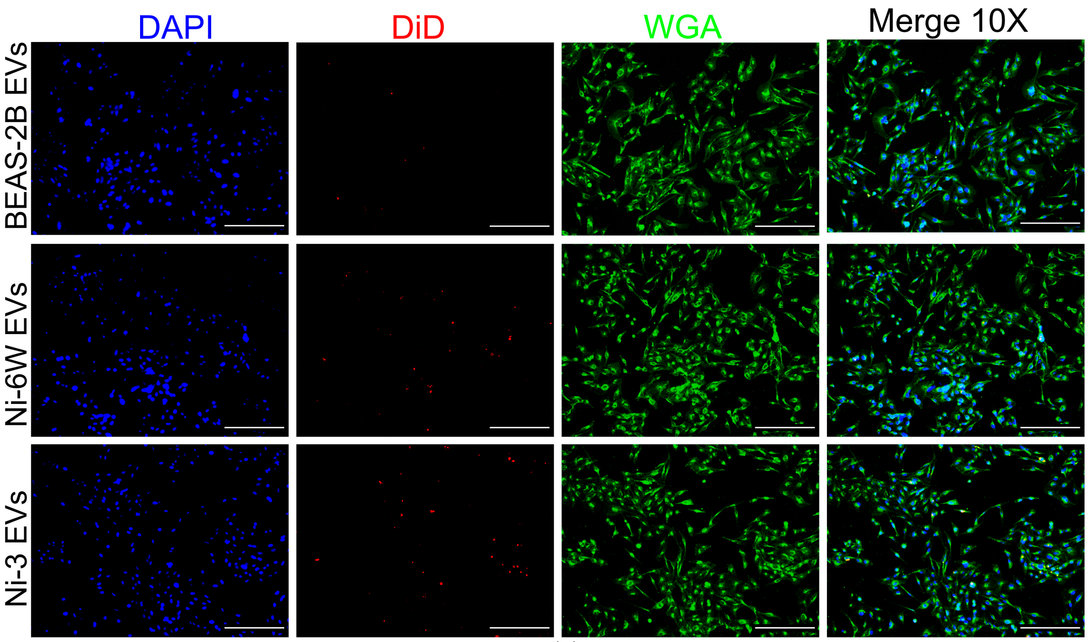

(**a**)

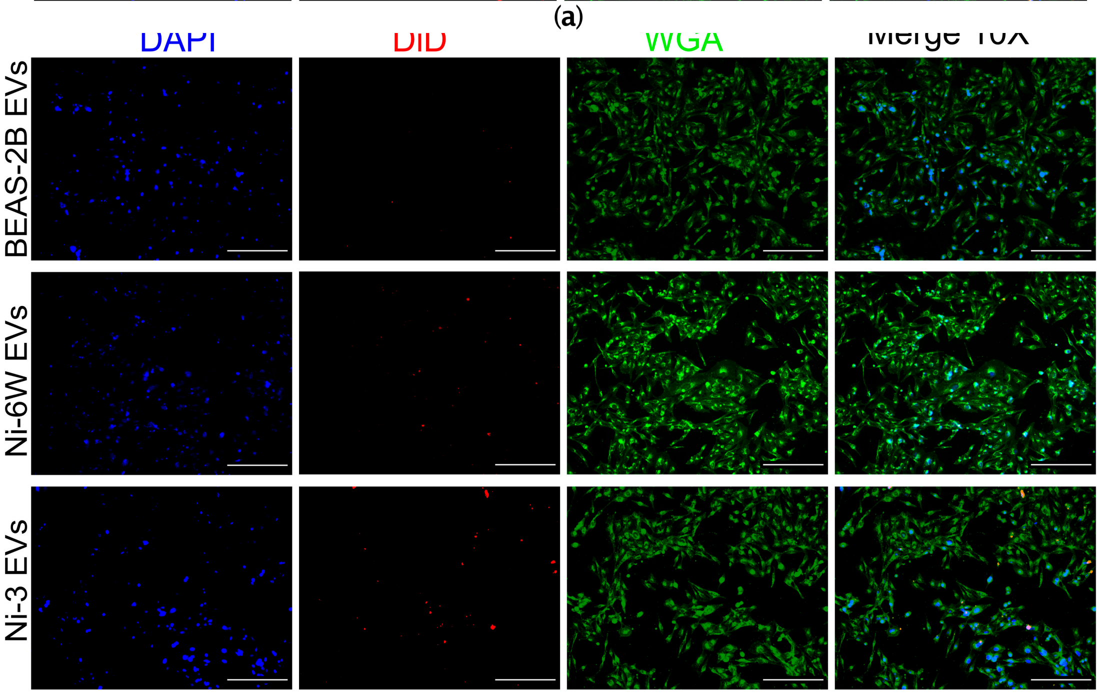

(**b**)

Figure S2. Uptake of EVs was affected by Ni exposure in BEAS-2B cells at 5 h. (**a**) EVs from Ni-6W, Ni-3 and BEAS-2B were isolated, labelled with DiD dye, and added to BEAS-2B cells for 5 h before being scanned with a Zoe fluorescence microscope (10X, scale bar: 400 μm). (**b**) BEAS-2B cells were pretreated with 100 μM Ni for 24 h before performing 5 h-EV-incorporation assay. Blue-DAPI, red-DiD, green-WGA, red arrow-incorporated EVs.

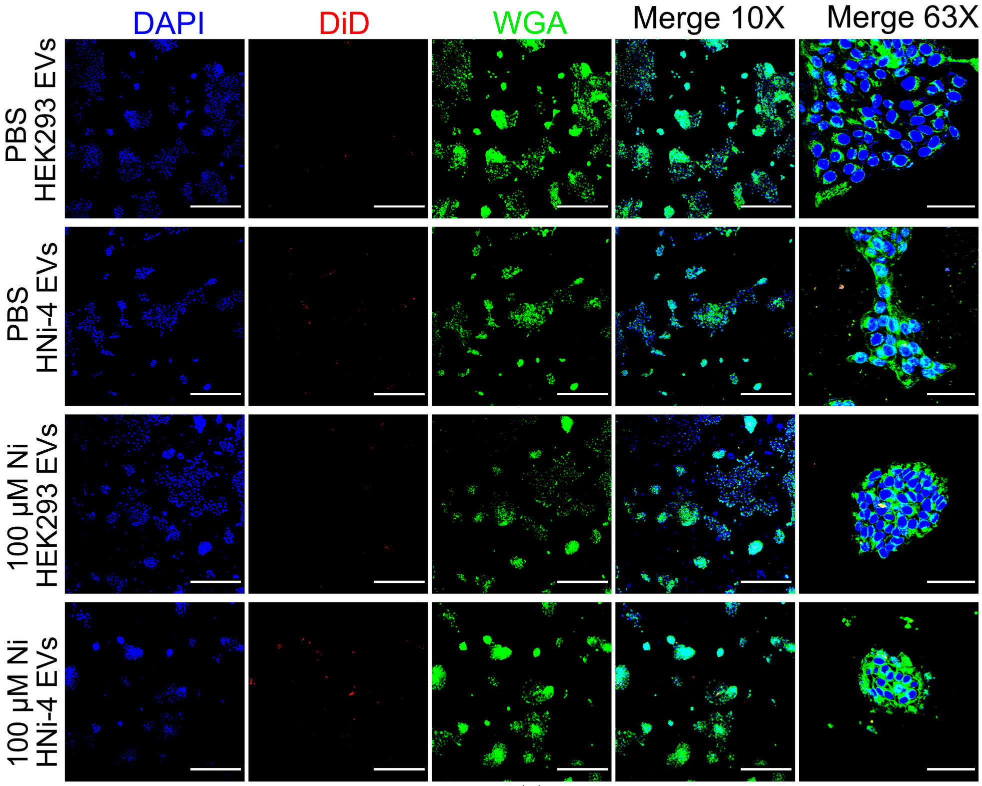

(**a**)

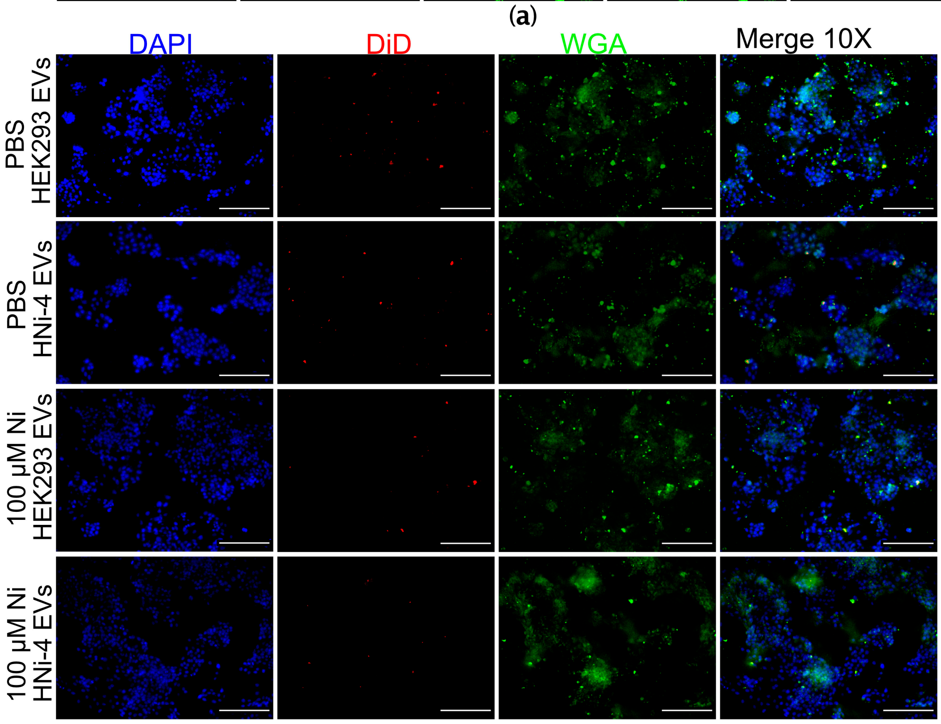

(**b**)

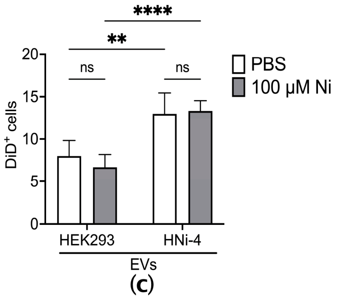

(**c**)

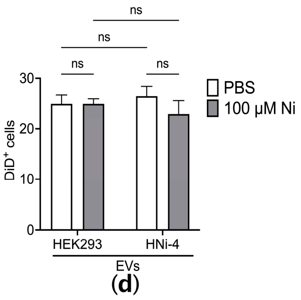

(**d**)

Figure S3. Uptake of EVs was affected by Ni exposure in HEK293 cells. (**a**) HEK293 cells were pretreated with

PBS or 100 μM Ni for 24 h before adding HNi-4 EVs or HEK293 EVs for treating 1 h. (**b**) HEK293 cells were pretreated with PBS or 100 μM Ni for 24 h before adding HNi-4 EVs or HEK293 EVs for treating 3 h. Blue-DAPI, red-DiD, green-WGA, red arrow-incorporated EVs. (**c**) and (**d**) Quantification and comparisons of the number of DiD-positive cells in (**a**) and (**b**), respectively. Scale bar: 400 μm (10X) and 60 μm (63X). Values are presented as mean ± SEM. **$p < 0.01$, ****$p < 0.0001$.

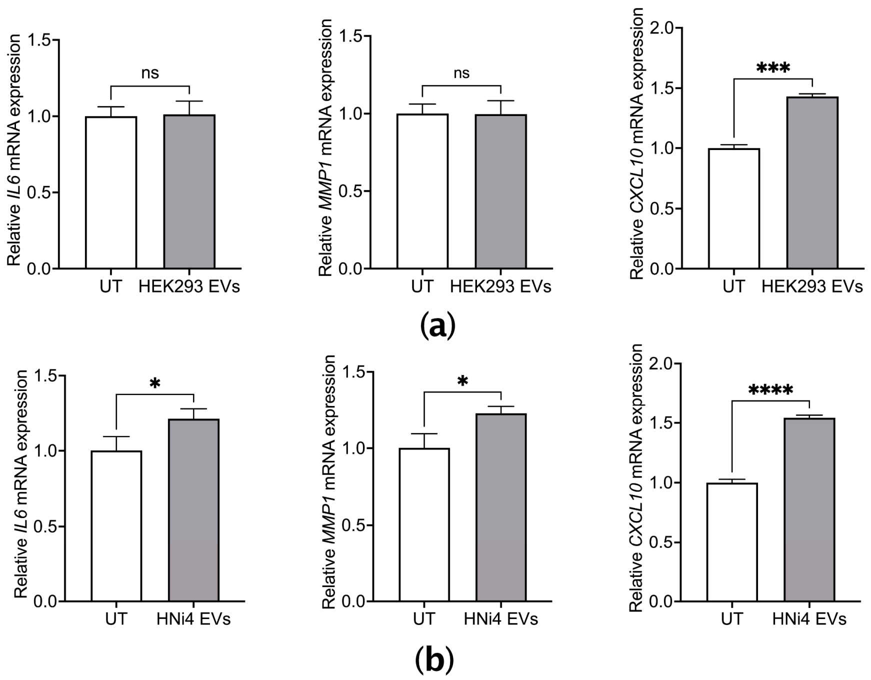

Figure S4. EVs from Ni-transformed cells induced mRNA expression of inflammatory markers in HEK293 cells. mRNA expression of inflammatory markers including IL6, MMP1 and CXCL10 were measured by quantitative real-time PCR after HEK293 cells were treated with 10 μg/mL of EVs from HEK293 (**a**) or HNi-4 (**b**). Values are presented as mean ± SEM. *$p < 0.05$, ***$p < 0.001$, ****$p < 0.0001$.

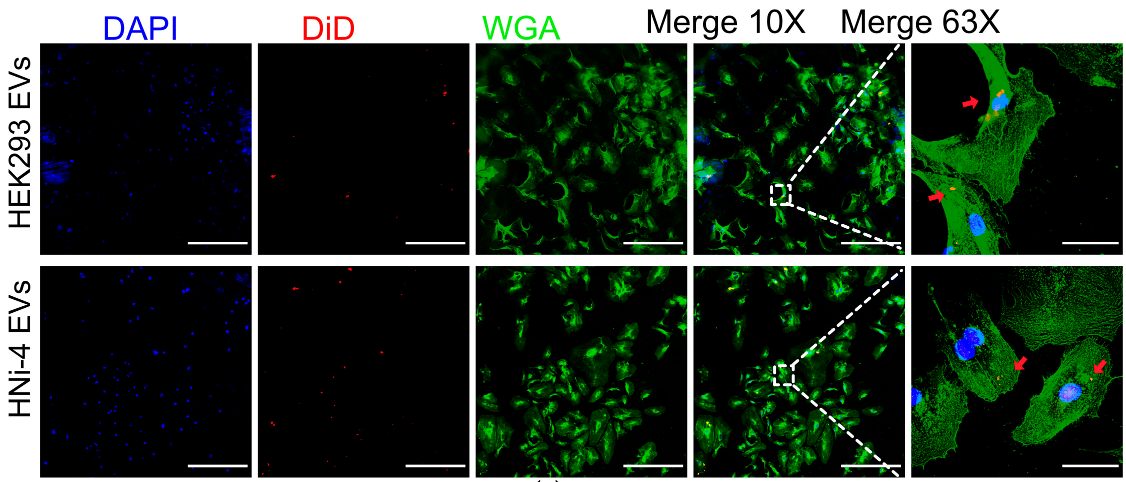

(a)

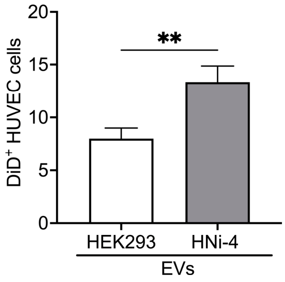

(b)

Figure S5. Ni exposure affected incorporation of HEK293 derived EVs in HUVEC cells. (**a**) EVs from HEK293 and HNi-4 were isolated, labelled with DiD dye, and added to HUVEC cells for 5 h before being scanned in confocal microscope (scale bar: 400 µm (10X) and 60 µm (63X), Zeiss 880). Blue-DAPI, red-DiD, green-WGA, red arrow-incorporated EVs. (**b**) Quantification and comparisons of the number of incorporated EVs in HUVEC cells. Values are presented as mean ± SEM. $^{**}p < 0.01$.

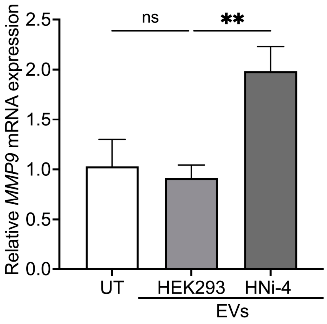

(a)

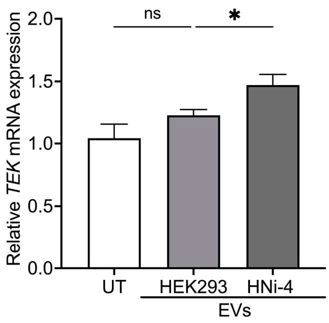

(b)

Figure S6. EVs from Ni-exposed HEK293 cells altered inflammation marker and coagulation marker in HUVEC cells. HUVEC cells were treated with 10 µg/mL of EVs from HEK293 or HNi-4 cells. mRNA expression of coagulation markers including (**a**) MMP9 and (**b**) TEK were measured by quantitative real-time PCR. Values are presented as mean ± SEM. $^{*}p < 0.05$, $^{**}p < 0.01$.
